# P27. Alemtuzumab (anti-CD52 monoclonal antibody) as single-agent therapy in patients with relapsed/refractory chronic lymphocytic leukaemia (CLL) – a single region experience on consecutive patients

**DOI:** 10.1186/2051-1426-2-S2-P18

**Published:** 2014-03-12

**Authors:** S Eketorp Sylvan, J Lundin, M Ipek, M Palma, C Karlsson, L Hansson

**Affiliations:** 1 Karolinska Institute and University Hospital Solna, Departments of Hematology and Oncology, Stockholm, Sweden; 2 Biostatistics, PAREXEL International, Berlin, Germany

## 

Alemtuzumab, a humanised CD52 monoclonal antibody, is routinely used as treatment for patients with refractory chronic lymphocytic leukaemia (CLL). Although alemtuzumab has been evaluated in numerous prospective clinical trials, little is known about its safety and efficacy in the routine clinical practice setting. Given that the haematology centers in the Stockholm area has gained substantial experience on alemtuzumab usage, starting already in the early 1990s, it would be of interest to compare our results obtained in routine health care with those from other reports including multicenter clinical trials.

Records from 1301 patients with CLL from the Stockholm-Cancer-Registry (1991-2010) identified 56 patients treated with alemtuzumab in the relapsed or refractory setting.

The median age was 69 years, 88% had advanced Rai stage with a median of 3 prior lines of therapy. One fourth had bulky lymphadenopathy and 73% were refractory to purine analogues. The median cumulative dose of alemtuzumab was 930 mg, being significantly higher (p=0.0277) for responders (1063 mg) compared to non-responders (643 mg). The median duration of therapy for responders was 12.4 weeks (range 7-32 weeks) and in all patients 11.6 weeks (range 1-51 weeks). The overall response rate (ORR) was 43%, with a response rate of 32.5%, 50% and 87.5% in the refractory, purine analogue relapsed and non-purine analogue relapsed group respectively. The differences in response rate was statistically significant (p=0.0104). A good performance status (PS) was associated with better response rate (ECOG 0-1 vs. ≥2) (p=0.0227). The median time to treatment failure (TTTF) was 7.8 months (range 0.4-55.4 months) being significantly longer for responders, 13.4 months (range 3.9-55.4 months) than for non-responders, 6.1 months (range 0.9-16.3 months) (p<0.0001). The median time to next therapy was 12.7 months (range 0.4-55.4 months).

Major infections (defined as ≥ grade III) occurred in 36% of the patients. Cytomegalovirus reactivation was the most common opportunistic infection (75%) occurring in 9 patients. Median overall survival was 22.5 months (range 0.4-74.3 months). Responders had a significantly longer survival than non-responders, 44.2 and 16.3 months respectively (p=0.0006, log-rank test), although the difference when using the Landmark method was not significant. Predictive factors for longer survival was PS grade 0-1 (p<0.0001) and fewer previous treatment lines (1-3 vs. ≥4) (p=0.0038, log-rank test). Twelve patients were retreated with alemtuzumab at least once with an ORR of 50% and a TTTF of 7 months (range 0.5-13 months).

In summary, a high cumulative dose/longer duration of therapy as well as relatively high response rates and long median survival was observed compared to what has been reported earlier in similar groups of patients having received alemtuzumab in trials or in routine health care in other areas. Optimal patient identification and management may result in avoidance of early discontinuation and possible better treatment outcomes.

